# Supplementary material for: Are carbon emissions trading and green financial instruments synergistic? -Comprehensive quantitative research based on content analysis
Source: PLoS One. 2024 Mar 7;19(3):e0298601. doi: 10.1371/journal.pone.0298601 (PMC10919601; doi:10.1371/journal.pone.0298601)
Supplement: S1 Data — (ZIP) [file pone.0298601.s001.zip › Data/地级市Z_F工作报告.zip!/╣π╢1⁄2/╢1⁄2▌╕╩╨/└┤╫╘╣1⁄2╓┌║┼í╛┬φ┐╦╩2╛▌═°í┐_2003.docx]

各位代表：

现在，我代表市人民政府向大会作政府工作报告，请予审议，并请政协委员和其他列席人员提出意见。

2002年工作回顾

过去一年，市政府在省委、省政府和市委的正确领导下，在市人民代表大会及其常务委员会和市政协的监督支持下，高举邓小平理论伟大旗帜，全面贯彻“三个代表”重要思想，以建设现代化中心城市为总目标、总任务统揽工作全局，抓住中国加入世贸组织的历史机遇，积极应对各种挑战，奋发进取，开拓创新，顺利完成了市十二届人大四次会议确定的各项任务。

——综合经济实力跃上新台阶。国民经济持续快速发展，多项指标在全省和全国的地位进一步提升。全市国内生产总值672.3亿元，总量排名全省第四，比上年增长18.4%，增幅名列珠三角各市首位；固定资产投资总额190.1亿元，增长51.5%；社会消费品零售总额225亿元，增长14.7%；地方财政收入55.3亿元，增长42.9%；年末金融机构本外币各项存款余额1902.4亿元，比年初增长21.5%，占全国的1.04%，其中城乡居民本币储蓄存款余额1001.7亿元，增长25.3%，占全国的1.15%；总供电量258.5亿千瓦时，增长24.5%。人民生活继续改善，城镇居民年人均可支配收入16949元，增长3.7%；农村居民人均纯收入7823元，增长6.2%。对国家的贡献越来越大，全年向中央和省上缴财政收入112.6亿元，增长29%；外来员工通过邮政汇出金额142亿元，增长3.2%。

——经济结构继续优化。2002年三大产业比例达到4.8:55.2:40。调整农业结构，扩大花卉、水果生产，实现农业总产值53.7亿元，增长6.8%，农业的经济效益和生态效益不断提高。调整工业结构，巩固了电子及通讯设备、电气机械及器材、纺织等产业的支柱地位，加快了高新技术产业的发展。全年工业总产值1574.1亿元，增长21.8%，其中高新技术企业产值占25.9%。全市省级高新技术企业总数达到102家，新增27家，是增加最多的一年。实施专利促进计划，全年专利授权达2680件，总量排名全省第三，同比增长53%。调整第三产业结构，加快发展物流、金融、会展、旅游、通讯、房地产等产业。金融运行良好，不良贷款率下降至10.1%，金融安全系数进一步提高，现金收支从大投放转为大回笼。全年举办各类商品展销会57个，总成交金额320多亿元，分别增长39%和7%。接待海内外来莞游客960多万人次，旅游总收入51亿元，分别增长21%和20.8%；新增星级酒店20家，全市三星级以上酒店达到53家，其中五星级的7家。邮电业务总量181.7亿元，增长48.3%。商品房销售收入29亿元，增长37%。实施名牌战略，全市首次有“步步高”和“金正”荣获全国驰名商标称号，“步步高”同时荣获国家名牌产品称号，新增加广东省著名商标11个，是原有总数的1.6倍。全年引进各类人才11.3万多人，相当于原有人才总数的28.5%，其中具有本科以上学历或中级以上职称的中高级人才占61.5%，有力地配合了全市的产业升级和结构调整。

优化所有制结构，全市公有、外资和民营经济各展其长、共同发展。公有企业深化改革，生产和效益明显回升。国有及国有控股经济完成工业总产值129.8亿元，增长39.3%，实现利润19.2亿元，增长95.9%。外向型经济快速发展。全年合同利用外资24.9亿美元，增长 25.9%；实际利用外资21.5亿美元，增长18.3%；外贸出口总额237.4亿美元，增长25%，占全国的7.29%、全省的20%，连续七年位居全国大中城市第三名。民营经济蓬勃发展。全市新注册个体工商户和私营企业5.3万户，增长40.6%，总数达到18.2万户；新增注册资金80.1亿元，增长46.3%。民营经济全年纳税25.3亿元，增长55.6%，占全市工商税收的16.7%。全市现有市级民营科技企业500家，其中省级民营科技企业89家，省级高新技术企业35家。

——市政府向市民承诺的十件实事基本完成。一是基础设施网络建设方面。完成了全市交通、绿地、供水等3项规划。新建改建了莞樟路、旗峰路等10条市区道路和望沙、樟谢、厚大等3条镇区联网道路，启动了五环路、东江大道、常虎高速、莞深高速三期、市长途汽车总站、公共汽车总站等工程。组建了东江水务集团公司，扩大了供水范围。积极争取省政府的支持，明确了我市对虎门港岸线、陆域和港政的管辖范围，理顺了虎门港与广州港的关系，加快了虎门港开发区的规划、建设和招商步伐。二是城市新区建设方面。编制了城市中心区等4项规划，完成了城市新区等2项形象设计；海关大厦等9项重点工程全部动工，其中展示中心和行政办事中心的主体已经封顶。旧区也整饰了万寿路等14条街道，新建了草塘公园、宏远西公园等8项绿化景观工程。三是松山湖科技产业园区建设方面。完成了约70公里的路基工程及大部分沥青路面的铺设，开工建设东莞理工学院新校区、生产力大厦等8项配套设施和北部科技工业园，引进20多个高新科技项目签约落户，中山大学生命科学学院、广东医学院等高校相继入园建设新校区，华南地区唯一的国家生命科学技术与人才培养基地也落户园区，松山湖被评为“中国最具发展潜力高新技术开发区”。四是整治环境污染方面。制定实施《东莞市水环境综合整治方案》，并与各镇区签订了责任书。建成市区污水处理厂首期日处理10万吨污水工程，去年10月投入运转，兴建东深沿线4镇生活污水处理厂，日处理260万吨污水的东莞运河樟村水质净化厂预计今年下半年投入使用。加强对重点污染源的监管，关闭查封污染工业企业12家，清拆养殖场375个，安装重污染企业自动监控装置105个，上路检测机动车尾气4460辆次，查处污染环境违法行为505宗。烟尘控制区扩大到237.6平方公里。清拆砖厂全面完成，整治采石场进展顺利，取得了良好的经济、社会和环境效益。五是市属企业改革方面。已有518户市属企业完成转制，占应转制企业总数的91%。严把资产评估关，实行承诺制、会审制、公示制，确保公有资产不流失。转制企业活力增强，职工基本满意，社会保持稳定。六是审批制度改革方面。在前年精简审批事项35%的基础上，去年再精简387项，减少48%，市级仅保留审批106项、审核122项、核准186项。颁发《东莞市行政审批管理办法》和《东莞市行政审批责任及过错追究办法》，规范了行政审批。七是电子政务建设方面。推广应用网上行政办公自动化系统，提高了行政效率。开通电子政务服务中心网站，整合全市政府部门的网站和网页，向市民提供政务公告、办事指南、表格下载、结果查询、咨询投诉等多项电子服务，已有20多万人次访问网站。八是农业农民农村工作方面。完成农村税费改革，全市农民负担减少7029万元。制定扶持欠发达地区发展的措施，加大帮扶力度。组织1亿元财政贴息贷款，扶持农业龙头企业11家。组织7000多万元财政贴息贷款，扶持7923个农户发展生产，有效解决了农民资金短缺的困难，降低了生产成本，增加了农民收入。拨款650万元，帮助684个困难户改造低标准住房，已建成1.37万平方米。安排专项资金600万元，向全市2695户低保对象的子女发放助学金。开展农村劳动力就业培训试点，取得较好效果。九是发展教育事业方面。东莞理工学院于2002年3月正式升格为本科院校，面向全省招生。按照全国1000所示范性普通高中的目标，高标准建设东莞中学、莞城一中、实验中学和高级中学。全市小学一年级开设英语课，小学四年级开设信息技术课，50岁以下的教师近80%取得多媒体计算机培训证书。整合职业教育资源，将农机学校并入理工学校。市教育信息网络中心、市直属学校校园网与省教科网实现光纤专线互联，全市有50所中小学基本建成校园网。全市幼儿在园率提高到94%，小学适龄儿童入学率达到100%，初中毕业生升学率达到91.1%，普通高中在校生与中等职业学校在校生的比例调整到1:1，每万名户籍人口中，职业高中和普通高中在校生的比例分别居全省第二和第三位。当年升读普通大学的比例达到每万名户籍人口44.7人，名列全省第二；高等教育毛入学率33.2%，达到现代化指标的要求，高等教育步入普及化阶段。德育工作继续加强，全市中小学生犯罪率为零。十是维护社会稳定方面。新增警察903名，加强装备建设，推进警务规范化，健全社会治安防范长效机制，加强流动人员管理，深入开展严打整治，防范和打击“法轮功”等邪教组织，全年刑事案件立案4.2万宗，同比下降6.3%，为如期实现两年治安形势明显好转打下了良好基础。健全三级劳动争议处理网络，依法调解、平息劳资纠纷1.2万多宗，维护了职工合法权益。整顿和规范市场经济秩序，全年共清理无证照经营4.4万多户，整治面达到80%左右。完善安全生产工作网络，落实责任制，有效遏止群死群伤重特大事故的发生，全市安全生产形势基本保持稳定。市镇两级政府建立健全控源治本的信访工作机制，有效减少了群众集体上访，全年越级到省上访人次同比下降10.9%。

——政风建设取得实效。去年是中央确定的“转变作风年”、“调查研究年”，我们大力推进政风建设，取得较好成效。深化政府机构改革，各部门简政放权，加强协调，推广“窗口式办文”、“一站式办公”，完善和落实绿色通道、并联审批、对外集中服务日、部门负责人协调会议等一系列服务措施。开展“依法行政、以德行政”教育，整顿执法队伍，加强立规工作，建立健全行政执法责任制，提高了执法和服务水平。推进政务公开，全市党政机关政务公开面达98%以上，村务公开面达100%。积极治乱减负，取消42项行政事业性收费，降低7项建设收费标准；三次调低售电价格，各类用户电价平均下降9.7%。健全“收支两条线”管理和单位内部财务管理，积极推进政府采购制度改革，全年市镇两级政府采购总额6.6亿元，比预算开支减少12.2%。成立会计核算中心、投资审核中心，强化财政监管。认真贯彻《党政干部选拔任用条例》，积极创建“好班子”，组织有关领导前往清华大学读书，举办世贸知识讲座，深入基层调查研究，开展全市村干部集中培训，进一步改进了领导作风，提高了干部综合素质。改进干部生活作风，严禁党员干部参与“黄赌毒”，严禁收送“红包”，制止铺张浪费。开展“机关作风万人评”，82个部门平均得分87.3，企业和群众对市级政府机关作风基本满意。

——政治建设和文化建设迈出新步伐。积极推进民主法制建设。坚持市长约见人大代表、会见政协委员制度，主动向人大汇报工作、向政协通报情况，自觉接受法律监督和民主监督。一年来市政府累计办理人大议案1件、建议101件，政协提案144件，做到了件件有回复。各职能部门向人大作专题汇报16项，职能部门负责人向人大述职3次，向政协通报情况18次。市政府制定规范性文件44件，提请市人大及其常委会审议通过规范性文件1件。加强基层民主建设，全市546个村委会和132个居委会顺利完成换届选举。

大力弘扬先进文化。认真学习邓小平理论和“三个代表”重要思想，深入贯彻党的十六大精神。广泛宣传《公民道德建设实施纲要》，积极开展“三现代”教育。组建中国歌舞团东莞分团，探索文艺发展新路子。加强文化设施建设和文物保护，茶山南社村古建筑群等9个单位列入第4批广东省文物保护单位。多种形式增建体育设施，广泛开展群众体育活动，创建了长安、常平、莞城罗沙等一批全国、全省体育先进乡镇和社区。积极发展竞技体育，在第11届省运会上，市体育代表团少年组团体总分名列全省第四位，成年组团体总分名列第十二位；全年莞籍运动员共夺得国际赛冠军2项、亚洲赛冠军4项、全国赛冠军35项、省赛冠军62.5项。改革医疗卫生体制，加强卫生监督和综合执法，规范医疗服务市场，促进卫生系统行风进一步好转。积极创建国家卫生城市，顺利通过全省第五次卫生城市检查评比。

——各项社会事业全面发展。社会保险扩面征缴稳步推进，全市离退休人员特别是企事业离退休干部的养老待遇得到提高，妥善处理了农民与职工基本养老保险的衔接。全面完成省下达的各项人口与计划生育指标，全市人口自然增长率为5.91‰。市医疗救济基金会发放资金641万元，救济了1123名贫困伤病患者。市法律援助处免费受理法律援助案件689宗，为受援人节省社会律师费206.7万元，解决了困难群众“打官司难”。积极帮扶欠发达地区，市镇两级支持广西河池、西藏、新疆和省扶贫基金会财物共计2276.5万元。妇女儿童发展规划实施良好。民兵预备役、双拥共建、国防动员、征兵和人防工作顺利开展。外事、侨务、对台、宗教、统计、打私、档案、方志、老龄和新闻出版等工作也都取得新的成绩。

各位代表！这些成绩，是在上级党委和政府以及市委的正确领导下，市政府和全体市民共同努力的结果，也离不开市人大、市政协的监督支持，离不开香港和澳门特区同胞、台湾同胞、海外侨胞、广大外商以及外来员工的关心支持，离不开驻莞解放军和武警官兵的帮助支持。在此，我代表市政府，向社会各界表示衷心的感谢！

在看到成绩的同时，我们也清醒地认识到，我市经济社会发展中仍然存在一些问题。主要是：产业结构仍不够合理，第三产业发展相对滞后，综合经济效益较低；城市化水平不高，城市的功能、形象、基础设施和文化氛围亟待改善，环境整治与生态保护任务迫切；发展不平衡，部分镇村经济相对落后，部分农民就业困难；行政管理体制与经济社会发展还有不相适应的地方；社会治安还不尽如人意；政风建设仍需进一步加强。对这些问题，我们将高度重视，在新的一年采取有力措施，切实加以解决。

2003年工作安排

党的十六大要求我们紧紧抓住新世纪头20年这个重要战略机遇期，全面建设小康社会，开创中国特色社会主义事业新局面。根据我市实际，市委制定了新世纪现代化建设“三步走”战略：2005年，全市国内生产总值超过1000亿元，比2000年翻一番，人均国内生产总值提前5年达到省提出的7000美元的目标；2010年，全市国内生产总值超过2000亿元，比2000年翻两番，人均1万美元，整体经济基本达到世界中等发达国家水平，率先基本实现社会主义现代化；2020年，力争全市人均国内生产总值比2010年再翻一番，达到2万美元，基本达到发达国家水平。

实现这个宏伟目标，是全市人民的迫切愿望，也是各级政府肩负的光荣使命。尽管我们还面临着许多问题与挑战，但经过20多年的改革开放，我们形成了产业配套齐全、基础设施发达、市场体制完善、投资成本较低、区域经济合作密切等相对优势，经济社会发展具有坚实的基础和巨大的潜力。我们对实现“三步走”目标充满信心。

今年市政府工作的指导思想是：坚持以邓小平理论和“三个代表”重要思想为指导，全面贯彻党的十六大和省委九届二次全会精神，落实市委十届七次全会的部署，以建设现代化中心城市为总目标、总任务统揽全局，按照“一网两区三张牌”的工作思路，以发展为主题，以结构调整为主线，以制度创新和科技创新为动力，以提高人民生活水平为根本出发点和归宿，推进城市、园区、环境和文化建设，努力建设经济强市，推动东莞向以国际制造业名城、文化新城和生态绿城为特色的现代化中心城市迈进。

今年全市经济社会发展的主要预期目标是：国内生产总值增长15%，地方财政收入增长15%，工业总产值增长18%，固定资产投资增长25%，社会消费品零售总额增长12%，实际利用外资增长6%，外贸出口增长10%，人口自然增长率控制在7.5‰以下，城镇居民人均可支配收入增长6%，农村居民人均纯收入增长5%。

为实现上述目标，要着重抓好以下八方面的工作。

一、调整产业结构，提高经济增长的质量与效益

走新型工业化道路，加快发展第三产业，推动东莞从加工基地向研发、制造、服务三位一体的国际制造业名城转变。

坚持以信息化带动工业化，以工业化促进信息化。大力发展电子信息产业，积极引进芯片设计、软件开发、数字技术、精密机械等“高、精、尖”项目。做好发展规划，积极申报东莞国家信息产业基地，争取早日获得国家认定。推进制造业信息化，建设综合性技术平台和一批行业性技术平台，引导企业在产品设计、制造、管理、营销、服务等全过程应用信息技术。鼓励、引导社会资本参与信息基础设施和各类应用系统的建设，加快信息化步伐。

依靠科技进步，推动工业结构升级。去年颁发了全市工业结构、信息产业结构和特色行业结构调整的实施方案，今年要继续抓紧落实。市财政安排专项资金，面向全社会选择有代表性的项目，组织实施技术创新项目和技术改造项目的导向计划，加快结构调整。做好电子信息、机械、医药、化工等4个行业的调研，按照高附加值、高技术含量、高关联度、低能耗、低污染的原则，指导结构调整。巩固信息产业的支柱地位，增强配套优势，延长产业链条。大力发展生物技术产业。加快发展装备工业。建设技术创新体系，主要面向电子信息产业和特色产业建立服务全行业的技术创新中心，加快共性技术的开发和推广应用，力争建立10个市级企业工程技术研究开发中心，组织一批企业申报省级企业研发中心。以松山湖科技产业园区为载体，搭建技术、人才、服务平台，吸引国内外科研单位、大专院校和大企业前来建立研发机构，提高本地科研水平。用好人才发展、留学人员和科研发展三项基金，加大人才引进力度，争取到年底全市拥有硕士研究生以上学历或高级职称的人才数量比2002年增加25%；实施“东莞市百名专家引进工程”，争取今年引进10--15名专家或学科学术带头人。充分利用与清华等26所著名高校的合作关系，推动产学研合作，带动全市高新技术产业的成长。坚持可持续发展战略，推广资源节约与综合利用技术，推行清洁生产。

大力实施名牌战略，发展壮大特色经济。落实《东莞市实施名牌战略联席会议工作制度》，制订全市名牌带动战略中长期规划和实施意见，建立名牌产品培育机制，扶持企业创名牌，依法保护商标和品牌，争取今年再增加一批国家级、省级著名商标和名牌产品。以厚街、常平、石龙等专业科技创新镇为示范，发挥政府对产业发展的宏观调控作用，明确“主业兴镇”的战略定位，培育在当地相对集中的行业，比如印刷业、玩具业、汽车零配件生产销售业等等，做大做强特色产业，建设一批专业城镇。

以现代流通业为突破口，加快发展第三产业。贯彻市政府《关于大力发展现代服务业的意见》，推动服务业加快发展、壮大规模、优化结构，从传统型向现代型转变、从以生活消费为主向以生产服务为主转变、从劳动密集型向知识密集型转变，为我市经济发展提供有力的产业支撑。面向国际物流、区域物流和市域配送，从基础设施建设、信息网络建设、对外开放、市场培育、行业管理和技术改造等方面入手，完善扶持现代物流业发展的产业政策。重点建设常平大京九物流园、虎门港物流园和松山湖物流园，带动全市现代物流业整合资源，加快发展。以虎门服装、厚街家具、大朗毛织、常平农副产品、樟木头粮食饲料等专业市场为龙头，培育和发展一批规模大、档次高、辐射力强的专业性批发市场。大力发展会展经济，把电博会、家具展、服交会等打造成名牌展会，并积极创造条件，提高毛织、汽车、灯饰、农副产品等展会的水平与影响力；壮大会展企业，完善展馆配套功能与经营机制，推动我市会展业向规范化、专业化、市场化迈进；促进会展与国际采购相结合，建立生产制造与国际采购互动的平台。推动连锁商业发展，以品牌专卖店、大型综合超市和仓储式商店等业态，形成市场网络，拓宽经营领域，扩大企业规模，整合资源，优化服务。加大金融监管力度，依法维护金融债权；改革农村信用社体制，积极筹建东莞市农村合作银行；扩大金融业开放，争取引入台资商业银行到我市设立代表处；加快银行之间业务系统的联网联合，建立全市“缴费一卡通”；积极解决民营企业物业办证难、贷款抵押难等问题，发展信用担保公司等中介组织，疏通民营企业融资渠道。贯彻《关于加快全市旅游业发展的意见》，放宽旅行社市场准入条件，支持大型旅游度假设施的建设，健全城市旅游服务功能，以商务会展、休闲度假和购物娱乐等为主要产品，以同沙生态公园、虎门威远岛和可园为重点景区，加大旅游资源的开发与推介力度，打造“魅力新东莞”的旅游新品牌。结合政府职能转变，将中介服务职能从政府部门转移到社会，大力发展法律、会计、审计、公证、代理、经纪、信息服务、科技服务、质量检测、工程咨询、资产评估、资信评估以及行业协会等各类社会中介机构，逐步形成执业公正、管理规范的中介服务体系。积极稳妥发展房地产业，有效启动市民住房消费，促进城市建设，培育新的经济增长点；坚持统一规划、总量控制、成片开发、综合配套的房地产开发政策，确保新开发小区有完善的教育、文化等公共配套设施；开展现状调查和社会供求研究，对房地产开发规模和供应结构进行宏观调控，防止超量供地和超前开发，注意发展经济适用房；加强市场监管，按照国家要求完善对商品房预售的管理，防止投机炒作和楼盘“烂尾”。

二、加快城市建设，优化城市功能与环境

加快城市化是东莞率先基本实现现代化的重要战略与迫切要求。我们要按照“拉开框架、优化布局、强化功能、改善环境、提高品位”的要求，推进城市的规划、建设、经营和管理，争取今年达到中国优秀旅游城市和国家卫生城市的创建标准，并为明年创建国家环保模范城市打下基础。

加快改善城市硬环境。按照“一年一大步，五年见新城”的要求，继续掀起新一轮基础设施建设高潮。加强与周边城市的沟通和协作，实现珠三角资源共享，优势互补，共同发展。交通及站场设施方面，深化全市交通规划，合理布局城际快速干线、市内高速公路和等级公路，抓紧建设常虎高速公路等工程，年内完成东江大道、松山湖快速干线、莞深高速东莞立交、寮步车检场等项目的建设，完成鸿福西路、鸿福东路、坝头路、圆岭路、东城南路、中心区路网等项目的改造。供排水方面，启动市区污水处理厂二期、截污主次管道二期、第五水厂二期、小区污水出户分流等工程，下半年完成樟村水质净化厂等工程。电力方面，盘活虎门电厂资产，加快建设500千伏莞城、横沥输变电工程，兴建一批220千伏和110千伏输变电工程，缓解我市电力供需矛盾。燃气方面，启动全市小区管道燃气工程等项目。

实施“南拓、北合、西进、东联”的城市发展战略，优化市域空间布局。新区方面，抓紧建设已铺开的9项重点工程，年内建成行政办事中心、展示中心、图书馆、科学技术博物馆、市区群众艺术馆、青少年活动中心、海关大厦、人民会堂等8项工程，启动并完成中心区广场、中心区环境完善、黄旗山城市公园首期等3项工程。旧区方面，依靠各区和民间投资，有计划地开展“穿衣戴帽”，改造“城中村”，扩建道路，增建公园、公厕、消防等公共设施，降低人口密度，改善人居环境，保护文化古迹，年内完成东江西岸岸线整治、运河两岸景观改造、坝头片区景观建设等工程。

改善城市软环境，创造优良秩序，提供优质服务。强化交通管理，协调有关部门联合整治交通秩序；增加投入，加快建设公安交通管理指挥中心和“电子警察”工程，提高执法效率；加快发展公共交通，增加公交运力和公共停车场，投放出租汽车，市中心区逐步取消摩托车营运；改善市内支干路网，规范交通标志，整治事故黑点；查处无牌无证车辆，禁止车辆乱停乱放，加强对运泥车、小四轮等营运车辆的监督管理，确保道路交通畅顺安全。

加强市容景观管理。清拆有碍市容市貌的各类临时建筑，坚决遏制违章乱搭建；禁止无序开山采石，已经开挖、影响景观的要及时复绿；整饰临街建筑立面，建设灯光夜景工程；增建城市雕塑和花园绿地，提高城市文化品位。在城市建设和道路改造时，搞好需求预测，协调有关部门一次性修好地下渠道，埋设各类线管，避免反复开挖道路。

抓好环境保护与城市卫生。建立镇区领导及有关负责人的环保责任考核制度。综合整治东江水环境，加快建设南畲朗和石马河治污工程，不让污水流入东江，确保饮用水源水质良好。抓紧编制全市生活污水处理厂建设规划，加快建设进度，由市东江水务集团公司牵头，以股份制方式建设和经营污水处理厂，投资比例市占50%，所涉及的有关镇区占50%。综合治理城区段运河，力争年底水质无黑无臭。结合产业结构调整和工业园区建设，加大力度，淘汰生产技术落后、环境污染严重的企业，推进重污染企业按行业集中建设、集中管理。凡位于城镇中心区、环境敏感区的污染企业，要加强管理，达标排放，到期的只能关闭或搬迁到相关工业园区，不许原地继续生产。开征垃圾处理费，制订垃圾处理厂建设规划，抓紧建设市区垃圾处理厂、危险废物处理中心，推动厚街、长安、清溪、横沥等镇建设垃圾处理厂。做好规划，分期分批整治复绿采石场。整治畜禽养殖业污染，扩大机动车尾气监测治理规模，所有医院的废水必须经治理后达标排放。加强建筑工地、集贸市场等地的卫生保洁，确保市容洁净。配合文明社区和国家环境保护模范城市的创建工作，在全市范围内广泛开展创建“绿色社区”活动，培养市民良好的环境保护意识与道德观念。

加强跨行政区域建设规划的协调，支持中心镇建设。积极创造条件，合并规模较小的镇村，优化整合资源。推进城市社区管理。结合村改居、居改社，健全社区运行机制，抓好社区居委会建设，发展社区教育文化卫生事业，完善社区服务。

三、扩大对外对内开放，发展外资、民营和内联经济

我们必须抓住入世过渡期的时机，在更大范围、更广领域和更高层次上参与国内外经济技术合作与竞争，以扩大开放来促进改革、推动发展。

坚持外向带动战略，发展外向型经济。改进招商方式，继续在国外设立招商办事处，加强宣传推介，积极开展上门招商、中介招商。进一步优化投资软环境，完善外资企业集中服务日、台商联络小组协调会议和大型企业绿色通道等制度，鼓励现有外资企业增资扩产，引进关联企业，设立研发机构。推行加工贸易网上审批新模式，积极配合海关开展快速通关、无纸通关、电子备案、深加工结转、电子核销和联网监管等各项改革。实施市场多元化战略，巩固港澳、欧美日、东南亚等传统市场，大力开拓俄罗斯、拉美、非洲、中东等新兴市场。坚持科技兴贸，逐步扩大拥有自主知识产权的高新技术产品出口，优化出口商品结构。大力发展一般贸易，加速市属外贸企业的转制，鼓励和支持各种不同经济成分的企业开展对外贸易。

推进工业进园，发展园区经济。重点做好松山湖、虎门港开发区和东部工业园的宣传推介，吸引更多的资金技术密集型大企业前来落户，带动全市招商引资。松山湖科技产业园区，是东莞对外经济技术合作的新载体、产业结构升级的龙头、产业支援服务业的中心，要按照东莞未来经济科技中心、综合性生态科技新城的目标高标准建设。今年上半年要全面完成征地拆迁和园区主干道路建设，年内争取完成行政办公区、商务区、国际学校等配套设施建设。抓紧建设松山湖大学科技城，建成东莞理工学院新校区和广东医学院东莞校区首期工程，9月对外招生，争取建成中山大学松山湖生物技术学院，积极吸引国内外其他有实力的著名高校进驻；年内完成松山湖生产力基地的初期建设，加快已签约各项目的实施，引进一批大型企业和中小科技项目入驻基地，推进产学研结合，为企业提供人才培训和技术支援，使大学科技城成为科技成果的孵化基地和产业化基地，推动园区发展。虎门港开发区要启动港口大道、立沙岛油气码头等建设项目。东部工业园要加快规划建设进度，争取今年上半年引进一批项目。

扶持发展民营经济，增强东莞经济发展后劲。鼓励发展民营科技企业、出口创汇企业和农产品加工企业，引导具备资格的民营企业申请外贸流通经营权和生产企业自营进出口权。支持民营企业组建行业协会或商会，加强合作交流与行业自律。鼓励民营企业参与国有企业资产重组，参与经营性基础设施项目的建设和营运。实施品牌战略，引导拥有自有品牌和自主知识产权的民营企业组建企业集团，实行规模扩张和集约经营，形成一批有实力的企业群。引导民营企业家守法经营，提高素质，转变经营理念，改变家族式经营方式，加快建立现代企业制度，逐步走上资本联合和科学管理的轨道，把企业越做越强，把品牌越打越响。

加强横向联合，发展内联经济。要加强与全国各地，尤其是香港、广州、深圳等周边城市的合作，推进建立珠江三角洲区域共同要素市场。鼓励外省市企业到我市投资办厂。根据市场经济规律和优势互补原则，结合产业转移、市场开拓等需要，组织企业等多种主体，采取多种方式，积极参加“大京九经济技术协作带”活动，对口帮扶韶关新丰、乳源两县和广西河池等欠发达地区。

四、深化各项改革，加快完善社会主义市场经济体制

党的十六大要求“改革要有新突破”，我们必须认真贯彻执行，与时俱进，深化各项改革。

继续深化公有企业改革。在基本完成市属企业产权制度改革后，抓好善后工作，指导改制企业建立和完善现代企业制度。切实加强对保留市属资本企业的管理，推行市属资本保值增值考核机制。按照管资产和管人、管事相结合的原则，继续推进市属资产管理体制创新，完成资产经营公司重组。全面加强市政府大宗物业和资源性资产的管理。

培育和完善现代市场体系。健全土地市场，对经营性用地实行公开招标拍卖，增加政府收益。增设建筑工程招投标市场分部，提高服务水平。完善市产权交易中心，争取银信部门抵债物业和镇区集体资产的处置都进入中心交易。加强对这三类要素市场的监管，查处违规行为，提高进场交易率。发展劳动力市场，打击非法职业中介。打破地方封锁和行业垄断，依法规范市场准入和竞争行为。加强部门协调配合，继续整顿无证照经营，严厉打击走私贩私、制假贩假、逃汇骗汇等各种破坏市场经济秩序的违法犯罪行为。不断健全完善市场信用体系。加强安全生产教育，落实安全生产责任制，深入开展重点行业专项整治，消除安全事故隐患。继续完善“一个窗口”收费等制度，整治乱收费，减轻企业负担，降低营商成本，增强投资环境的竞争力。

深化财税、分配、投融资等体制改革。贯彻落实“加强征管、堵塞漏洞、惩治腐败、清缴欠税”的方针，建立健全税收征管新模式，强化依法治税，加大稽查力度，打击各种偷税、骗税和逃税行为，促进财政收入稳步增长。深化“收支两条线”管理改革，推行综合预算，把所有部门资金一律纳入财政管理。抓好城市有形资源、无形资源收益的管理，确保政府资源性收入统归财政。尽快建立规范、高效的市属国有资产收益征管模式，避免资产流失，增加财政收入。改革政府采购制度，编制采购预算，分离采购与监管部门。凡市财政投资的建设项目，都要实行会计委派制度。严格执行投资项目评审办法，做好工程概算、预算、结算和竣工财务决算。完善按劳分配为主体、多种分配方式并存的分配制度，坚持劳动、资本、技术和管理等生产要素按贡献参与分配的原则，坚持初次分配注重效率、再分配注重公平的原则，加大对欠发达地区财政转移支付力度，提高社会保障水平。改革投融资体制，建立多元化的投资渠道，采取政府、民营、外商投资、股份经营、BOT（建设—经营—转让）等多种方式投资城市建设。

五、做好“三农”工作，实现农业发展、农民增收、农村稳定

全面建设小康社会的重要目标之一，就是做好“三农”工作，逐步缩小工农差别、城乡差别和贫富差别。

推进农业产业化，提高农业现代化水平。合理规划调整种养业的布局，建设一批结构调整示范点，重点引导沿海水乡地区农民优化种养结构，大力发展地方特色产品和区域优势产业。加强对已认定的11家市级农业龙头企业的动态管理，引导它们建立现代企业制度，不断完善产业化经营机制。以农产品流通加工型企业为重点，争取再培育一批市级农业龙头企业。加快建设农产品专业批发市场，重点发展市花卉博览中心、市（万江）果菜副食交易市场、石碣蔬菜批发市场和漳澎香蕉交易市场等。

落实优惠政策，扶持欠发达地区发展。市已经从8个方面制订了22条扶贫政策，扶贫工作领导小组及其办公室要加强综合协调，认真督促检查，确保优惠政策及时到位、扶贫资金专款专用、结对帮扶扎实深入。欠发达地区的各级干部要开拓创新，艰苦奋斗，总结经验教训，找准发展路子，用好用足市的扶贫政策，有计划、有步骤地推进脱贫奔康的各项工作。

加强镇村集体资产和财务管理。理顺镇级集体资产管理机构。抓紧出台《东莞市农村集体经济审计办法》。建立农村集体资产季度分析报告制度，加强对集体资产营运的监管。认真贯彻新修订的《东莞市农村集体资产管理实施办法》，坚持量入为出，强化计划管理，提高资产营运水平；严格控制开支，减轻集体债务；厉行勤俭节约，反对铺张浪费，规范集体分配，促进农村集体经济健康发展。

努力增加农民收入，提高农民生活水平。加快经济发展，广开就业门路。加强教育引导，积极转变农民就业观念，逐步实现农民福利与就业挂钩。充分利用现有教育资源，建立覆盖全市各镇区的职业技术培训网络，做好职业中介服务，提高我市劳动就业服务水平。认真落实农村税费改革方案，确保农民负担减轻，确保农村义务教育正常发展，确保农村社会稳定。因地制宜，多种形式建立农村合作医疗保障制度，力争覆盖率达到80%，确保农民享有较高水平的医疗保障。

六、提高社会管理水平，优化公共服务

加强社会管理，优化公共服务，是营造良好发展环境的重要途径。

积极稳妥地推进“村改居”。在市区4个街道办事处和7个中心镇，具备条件的村委会都要改为社区居委会。稳步推进多种形式的农村股份制，改革股权配置，完善经营机制、分配激励机制和监督约束机制，明晰集体资产产权，规范集体经济运作，妥善解决集体资产、农民宅基地、计划生育、福利分配特别是“出嫁女”福利分配等问题，维护社会稳定。同时转变农村基层组织职能，实行政企分开。

提高社会治安管理水平。坚决扫除“黄赌毒”和有组织犯罪活动，严厉打击“法轮功”邪教活动。强化社会面控制，进一步完善打、防、控一体化的治安防范网络，继续开展严打整治斗争，抓好流动人口的管理，完善警务责任区建设，提高警队素质。积极排查不稳定因素，依法妥善处置群体性事件。

加强人口与计划生育工作。提请市人大常委会修订《东莞市人口与计划生育管理规定》，全面推进依法管理。深入开展“婚育新风进万家”等宣传教育活动，强化基层基础工作，积极稳妥地推进计划生育村（居）民自治，实行工作重心下移。加强对流动人口计划生育的管理与服务，建立以现居住地管理为主的工作机制。充分发挥相关单位的职能作用，对人口与计划生育工作齐抓共管。坚决实行“一票否决”制，落实人口与计划生育目标管理责任制，稳定低生育水平。

构建现代化市政管理模式。按照政企分开、企事分开的原则，改革市政管理体制和运营体制。供水、燃气、污水处理、垃圾处理等公用行业经营性项目的建设，都要全面推向市场，鼓励社会资金、国外资本采取独资、合资等多种形式，进行投资、建设和运营，努力实现社会化管理、市场化运作和企业化经营，推动市政管理的现代化。

七、推进民主法制工作，建设社会主义政治文明

从制度建设和机构改革等方面加大工作力度，扩大社会主义民主，健全社会主义法制，巩固和发展民主团结、生动活泼、安定和谐的政治局面。

转变政府职能。积极建设“学习型、创新型、服务型”政府，切实把职能转变到经济调节、市场监管、社会管理和公共服务上来，解决好政府越位、缺位和错位的问题，提高驾驭市场经济的能力。坚持依法行政，加强执法队伍建设，落实行政执法责任制。继续深化行政审批制度改革，组建市政府行政审批服务中心，推进网上行政审批，先在与市民和企业联系比较密切的外经贸、劳动、工商等部门进行网上备案制审批的试点，再逐步过渡到联合办公和联合审批。积极稳妥地推进事业单位机构改革。

加快电子政务建设。继续完善信息化基础设施建设，形成高速、互联、安全的政务专网平台和对外服务平台。综合开发利用政府和社会各方面数据资源，形成面向全社会的公共平台。开展网上预审、协作办公和“一站式”电子政务的试点。

深入推进政风建设。贯彻执行十五届六中全会提出的“八个坚持，八个反对”的要求，切实改进思想作风、工作作风、领导作风和生活作风。选择一些部门和镇区开展政风建设试点，重点解决办事难、依法行政不到位、过错追究不落实、队伍素质不够高等问题，取得经验再逐步推广。继续开展“机关作风万人评”活动。政府公务人员要“干净干事”，廉政勤政，艰苦奋斗，厉行节约，严禁用公款吃喝玩乐、挥霍浪费，禁止收送“红包”和参与“黄赌毒”。

加强民主法制建设。健全民主制度，丰富民主形式，扩大公民有序的政治参与，保证人民依法实行民主选举、民主决策、民主管理和民主监督，享有广泛的权利和自由，尊重和保障人权。坚持市长约见市人大代表、会见市政协委员制度，积极拓宽人大代表、政协委员直接反映意见的渠道。支持各民主党派和工会、共青团和妇联等人民团体依照法律和各自章程开展工作。不断扩大基层民主，完善村民自治，健全村党支部领导下的村民自治机制；完善城市居民自治，建设管理有序、文明祥和的新型社区；完善职工代表大会和其他形式的企事业民主管理制度，保障职工的合法权益。广泛深入地开展“法德教育”，提高机关工作人员的法律和道德素质，拓展和规范法律服务，积极开展法律援助，加强对执法活动的监督，提高执法水平，营造良好的法制环境。

八、发展社会主义先进文化，建设社会主义精神文明

全面建设小康社会，必须大力发展社会主义先进文化，建设社会主义精神文明。

加强公民思想道德建设。深入学习党的十六大精神，认真贯彻《公民道德建设实施纲要》，加强社会公德、职业道德和家庭美德教育，广泛开展群众性精神文明创建活动，深入开展“三现代”主题教育，倡导科学、文明、健康的生活方式和行为习惯，争创广东省文明城市。

发展科技和教育事业。增加投入，创新机制，整合资源，优化结构，提高效益，积极创建教育强市。今年要有5至6个镇区建成省教育强镇，争取到2005年70%的镇区成为省教育强镇。加快市属中学布局的调整，建设市区3所中学、1所小学，东莞中学和实验中学争取今年申报国家级示范性普通高中。加快建设英语和信息技术教育特色工程，进一步推进素质教育。提高标准，扩大规模，继续推进联合办学。认真贯彻“积极鼓励、大力支持、正确引导、加强管理”的方针，壮大民办教育。把职业教育与民营经济和特色产业的发展结合起来，大力培养具备先进适用技能和创新创业精神的本地人才。高等教育要创新办学体制，优化人才培养的结构与效益，积极引进拔尖人才，承接高新技术研发项目和推动科技成果应用转化，增强服务地方经济发展的主动性。加快教育信息网络建设，进一步完善校园网，推进教育信息资源共享。鼓励科技创新，大力营造科技发展和人才成长的良好环境。加强科普教育工作，努力在全社会形成崇尚科学、反对迷信、健康向上的社会风尚。

繁荣社会主义文化，充实东莞文化底蕴。加强文化基础设施建设，重点抓好新城区图书馆、大剧院等工程和东莞美术馆的规划建设。充分利用现有文化设施，积极开展群众文化活动。今年下半年举办第二届东莞文化艺术节，坚持专业与业余相结合、提高与普及相结合、高雅与通俗相结合的原则，推出一批文艺精品，检阅全市文艺队伍，打造东莞文化品牌。坚持繁荣与整顿并举，培育和发展健康有序的文化市场。继续推进文化体制改革，大力发展文化公益事业和文化产业。借鉴中国歌舞团东莞分团创建经验，筹划成立东莞文学院、东莞画院等文艺团体。加强广播电视基础设施建设，推进全市广电网络的整合与升级。积极发展体育事业，增建群众健身场地设施，健全群众体育组织，举办经常性群众体育活动，积极开展“体育先进社区”争先创优活动。调整布局，扬长避短，有选择地发展竞技体育项目，培养体育人才，增强竞技体育实力。推动体育产业化，引导群众进行健康的体育消费。

发展医疗卫生、社会保障等各项事业。积极推进医疗卫生事业改革，加快建立和完善医疗卫生服务体系。创新办医理念，探索多种体制的办医模式，积极引导外资和民资开办医疗机构，促进医疗服务的公平竞争，提高服务水平。规范医疗市场，维护医疗安全。落实企事业单位离退休干部待遇调整政策，继续巩固养老保险与医疗保险同步扩面征缴的成果，开展企业补充养老保险，稳步推进失业保险扩面征缴，继续完善生育保险和工伤保险，全面推动社会保险事业发展。不断完善社会救助制度，切实保障困难群众的基本生活。抓好民兵预备役、国防教育、国防动员、双拥共建和人防工作，巩固军政军民团结，争取第五次荣获“全国双拥模范城”称号。落实有关政策，做好宗教、对台、外事侨务等工作。

各位代表！东莞面临着前所未有的发展机遇。让我们以邓小平理论和“三个代表”重要思想为指导，全面贯彻党的十六大精神，继续保持谦虚谨慎、不骄不躁的作风，继续保持艰苦奋斗的作风，与时俱进，开拓创新，同心协力，扎实工作，为全面建设小康社会，率先基本实现社会主义现代化而努力奋斗！
